# Neurological Outcome and Respiratory Insufficiency in Intramedullary Tumors of the Upper Cervical Spine

**DOI:** 10.3390/medicina59101754

**Published:** 2023-09-30

**Authors:** Kateryna Goloshchapova, Maria Goldberg, Bernhard Meyer, Maria Wostrack, Vicki M. Butenschoen

**Affiliations:** Department of Neurosurgery, Klinikum rechts der Isar, Technical University of Munich, 81675 Munich, Germanyvicki.butenschoen@tum.de (V.M.B.)

**Keywords:** intramedullary spinal cord tumours (IMSCT), upper cervical spine, neurological deterioration, respiratory insufficiency

## Abstract

*Background and Objectives:* Intramedullary spinal cord tumors (IMSCT) are rare entities. A location in the upper cervical spine as a highly eloquent region carries the risk of postoperative neurological deficits, such as tetraparesis or respiratory dysfunction. Evidence for respiratory dysfunction is scarce. This study aimed to describe these highly eloquent tumors’ early and late postoperative clinical course. *Materials and Methods:* This is a single-center retrospective cohort study. We included 35 patients with IMSCT at levels of the craniocervical junction to C4 who underwent surgical treatment between 2008 and 2022. The authors analyzed the patients’ preoperative status, tumor- and surgery-specific characteristics, and follow-up functional status. *Results:* The study cohort included twenty-two patients with grade II ependymoma (62.9%), two low-grade astrocytomas (5.7%), two glioblastomas (5.7%), six hemangioblastomas (17.1%), two metastases (5.7%), and one patient with partially intramedullary schwannoma (2.9%). Gross total resection was achieved in 76% of patients. Early dorsal column-related symptoms (gait ataxia and sensory loss) and motor deterioration occurred in 64% and 44% of patients. At a follow-up of 3.27 ± 3.83 years, 43% and 33% of patients still exhibited postoperative sensory and motor deterioration, respectively. The median McCormick Scale grade was 2 in the preoperative and late postoperative periods, respectively. Only three patients (8.6%) developed respiratory dysfunction, of whom, two patients, both with malignant IMSCT, required prolonged invasive ventilation. *Conclusions:* More than 60% of the patients with IMSCT in the upper cervical cord developed new neurological deficits in the immediate postoperative period, and more than 40% are permanent. However, these deficits are not disabling in most cases since most patients maintain functional independence as observed by unchanged low McCormick scores. The rate of respiratory insufficiency is relatively low and seems to be influenced by the rapid neurological deterioration in high-grade tumors.

## 1. Introduction

Intramedullary spinal cord tumors (IMSCT) are rare entities and account for only 5–10% of all spinal tumors. The most frequent entities are ependymomas, astrocytomas, and hemangioblastomas [1]. Patients with low-grade tumors usually present with mild neurological deficits such as gait ataxia, hypesthesia, or pain. The optimal treatment for IMSCT depends on the tumor type. For most IMSCT, the standard therapy is the surgical gross total resection (GTR), although it bears a risk for neurological deterioration [2,3]. The GTR is the main predictor of progression-free survival in benign or low-grade entities [4]. The GTR results in a more prolonged progression-free survival with an excellent neurological long-term outcome for ependymomas, hemangioblastomas, and pilocytic astrocytomas [3]. However, for high-grade astrocytomas, surgical resection does not provide any oncological benefit, is often not feasible, and comes along with a high rate of neurological impairment; therefore, usually a biopsy with adjuvant chemo- or radiotherapy is the commonly accepted approach [5].

The most commonly affected site of IMSCT is the cervical spine [3]. Especially their location in the upper cervical spine, i.e., the craniocervical junction downwards, up to the level of C4 or C5, carries the highest risk for postoperative neurological deficits resulting in a tetraparesis—a devastating disability condition. Additionally, surgical treatment may result in respiratory failure due to the innervation of the diaphragm by the C3-5 nerve roots. Further, tumor mass extending into the medulla oblongata can aggravate respiratory dysfunction. This problem is commonly known in patients who experienced traumatic spinal cord injury [6]. However, there is little evidence of this complication in patients who undergo surgery for IMSCT. We are hereby investigating the surgical risks of intramedullary tumor surgery in the upper spine with particular emphasis on respiratory failure.

## 2. Patients and Methods

We conducted a retrospective cohort study of patients who underwent surgical resection of IMSCT above or at the C4 level from January 2008 to December 2022 at our tertiary care institution.

### 2.1. Surgery Protocol and Postoperative Management

Microsurgical techniques with continuous neuromonitoring were used in all cases. Patients were placed in a prone position with a rigid head fixation in the Mayfield clamp. The most common approach was laminoplasty (57%), followed by a durotomy and median myelotomy in most cases. For tumors near the surface of the spinal cord, we used either the transpial method or the dorsal root entry zone technique. Tumor debulking using a cavitron ultrasound surgical aspirator (CUSA) was applied in more extensive tumors (excluding hemangioblastomas) before the final tumor removal to minimize traction damage to the spinal cord tissue. We did not use intraoperative ultrasound for imaging purposes.

The local multidisciplinary tumor board decided on surgical and adjuvant treatment. High-grade astrocytomas received fractional radiation therapy with a total 40–60 Gy dose in 1.8 to 2-Gy fractions with or without Temozolomide therapy. Patients with ependymomas and hemangioblastomas underwent follow-up examinations every 12 months.

### 2.2. Medical and Surgical Data

All patients had preoperative contrast-enhanced magnetic resonance imaging (MRI) of the spinal cord to evaluate the tumor location, size, and extent. The tumor volume was calculated using the formula for ellipsoid: 4/3 × π × ABC/2, where A, B, and C are the length of semi-axes. We also assessed the presence of syrinx on the preoperative MRI scan.

The surgery-specific information included the extent of resection (GTR defined as complete removal of the contrast-enhancing tumor, subtotal resection (STR) defined as removal of more than 90% of the contrast-enhancing tumor, tumor debulking, or biopsy), as well as the intraoperative changes in recordings of the intraoperative neuromonitoring. The surgical approach was subclassified as unilateral (interlaminar fenestration or hemilaminectomy) or bilateral (laminoplasty and laminectomy), depending on tumor extent and tumor location within the spinal canal.

In our cases, tumor histology was defined according to the current WHO classification, congruent with the WHO 2021 classification. The preoperative neurological status was evaluated. The outcome was assessed at discharge and the most recent follow-up. We used the modified McCormick scale to determine the clinical status of the patients, which is a widely accepted tool for neurological grading function in spinal cord tumors [7]. We also recorded any postoperative neurological worsening, such as new motor deficit defined with the Medical Research Council classification (MRC), hypesthesia, aggravation of gait ataxia, or respiratory insufficiency, which required postoperative intubation, prolonged weaning, or respiratory tract infections.

The patients underwent a follow-up MRI three months after the surgery and had yearly imaging and clinical examinations afterward as long as their clinical and neurological status remained stable. In cases of hemangioblastoma, further examinations to rule out Von Hippel-Lindau syndrome were conducted.

### 2.3. Statistical Analysis

Statistical analyses were performed using Microsoft Excel (Version 2019) and StatTech v. 3.1.6. (Developer—StatTech LLC, Moscow, Russia). The median or mean values were compared using the Student *t*-test. The correlation between potential predictive factors and the transient and permanent neurologic deficit was analyzed with a chi-square test for the categorical variables such as sex, the extent of resection, presence of syrinx, WHO grade, tumor entity, and preoperative McCormick scale. For continuous variables such as tumor volume, age, and duration of symptoms, the Kruskal–Wallis test was utilized.

## 3. Results

### 3.1. Entities and Presentation

We included 35 consecutive patients who underwent a surgical treatment protocol; 57% of patients were male with a mean age of 45 ± 16 years. The mean duration of symptoms before the intervention was 22 ± 48 months. Patients with high-grade tumors (metastasis and high-grade astrocytomas) had a significantly shorter symptom duration than those with low-grade counterparts (*p* = 0.012). The median follow-up period was 3.27 ± 3.83 years and was available for 29 patients (83%).

The most common type of tumor was ependymoma WHO°2, which affected twenty-two patients (62.9%), followed by astrocytoma WHO°2–4 in four patients (11.4%), hemangioblastoma WHO°1 in six patients (17.1%), and carcinoma metastasis (adenocarcinoma of colon cancer and melanoma metastasis) in two patients (5.7%). Four patients (11.4%) had a clinical diagnosis of neurofibromatosis type 2. Two patients (5.7%) had recurrent tumors at the same level, and two patients (5.7%) had drop metastasis of ependymomas at different levels from the upper cervical spine (Table 1).

The tumor extension ranged from 1 to 9 segments, with a mean of 2.8. The mean tumor volume was 1.86 ± 2.58 cm^3^. Syringomyelia was found in eight out of thirty-five patients (23%). Table 2 shows the initial clinical symptoms that prompted the diagnosis. Most preoperative symptoms were mild, whereas 29 of 35 patients (83%) had a grade 1 or 2 on the modified McCormick scale rendering them functionally fully or nearly independent. Only five patients harboring high-grade astrocytomas or carcinoma metastases had a rapidly progressive neurological deficit.

### 3.2. Surgical and Clinical Outcome

GTR was achieved in 77% of patients who were candidates for this procedure because of the suspected tumor biology. In three patients, we performed a biopsy followed by resection in one case and combined radiation and chemotherapy in two cases with confirmed high-grade gliomas.

**Figure 1:**
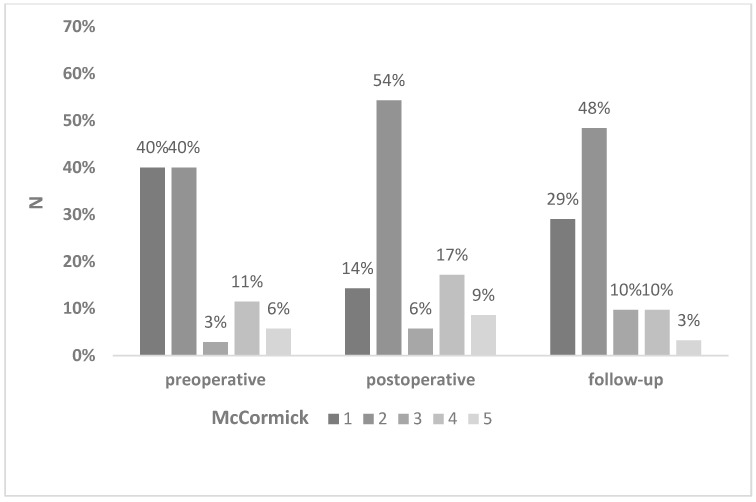
Bar graph showing preoperative, postoperative, and follow-up functional status of patients according to the modified McCormick scale.

New dorsal column-related deficits, i.e., sensory loss and gait ataxia, and motor deficits occurred in 64% and 44% of patients, respectively (preoperatively 38.9% motor deficits, Table 2). At follow up, 25% of patients with dorsal column-related dysfunction and 33% with motor dysfunction recovered to at least their preoperative status. Permanent new motor deficits were observed in 31%, and sensory and gait dysfunction in 43% of patients, respectively. In two patients (5.7%), the preoperative neurological symptoms improved after surgery, but 40% of the cases were clinically independent before surgery and therefore had no possibility of objective clinical improvement. Three patients had further neurologic decline during the follow-up, related to the tumor progression in one case and neurofibromatosis type 2-associated secondary ependymoma manifestation in two cases. The most common deficits were hypesthesia and gait ataxia (Table 3). The results of preoperative, postoperative, and follow-up McCormick scores are summarized in Figure 1. The median preoperative and follow-up McCormick score was 2, with no significant difference in the preoperative and follow-up functional status (*p* = 0.0897).

### 3.3. Respiratory Dysfunction

Three patients (8.6%) experienced respiratory complications in the early postoperative course. Two patients with high-grade lesions (glioblastoma WHO°4 and intramedullary metastasis) required prolonged mechanical ventilation and subsequent tracheotomy after the failed weaning. Both of these patients had already presented with higher-graded tetraparesis before surgery. One of these patients was successfully decannulated during the follow-up period of 3 months; the other died during the same hospital stay as the treatment plan was switched to palliative care due to poor prognosis of the melanoma metastasis of the craniocervical junction. A third patient developed respiratory failure mainly due to a bronchial obstruction by a mucus plug. None of the remaining 32 patients (92%) developed any signs of respiratory dysfunction, including pneumonia or dyspnea.

It is possible that respiratory complications and deficits may have occurred after the hospital stay but were not documented. These could include subclinical aspirations, which did not lead to pneumonia requiring antibiotics, desaturation, or tachypnea. Therefore, it is likely that the percentage of C4 paresis has been underestimated in this case.

In addition to the initial three patients mentioned, another trio underwent chest X-rays in order to pinpoint the source of their elevated infection levels. However, none of these individuals exhibited any signs of diaphragm (hemi-)paresis.

Other surgery-related complications included postoperative hemorrhage that required surgery to remove the hematoma in one case and delayed wound healing in two patients who underwent revision surgery. The overall perioperative complication rate, including patients with a postoperative respiratory failure, was 14%. The 30-day mortality was 2.8% (*n* = 1), whereas this case was not surgery-related.

The preoperative McCormick scale and the WHO grades were the only predictors of the transient (*p* < 0.001 and *p* = 0.045, respectively) and permanent postoperative impairment (*p* < 0.001 and *p* = 0.009, respectively) (Table 4; Table 5). There were no statistically significant differences when comparing postoperative and follow-up McCormick scores on demographic parameters, duration of symptoms, tumor size, the presence of syringomyelia, or surgery-specific data. Tumor recurrence and operations at multiple spinal levels in NF patients had an unfavorable clinical outcome compared with patients without tumor recurrence and single lesions.

### 3.4. Case Presentation

**Figure 2:**
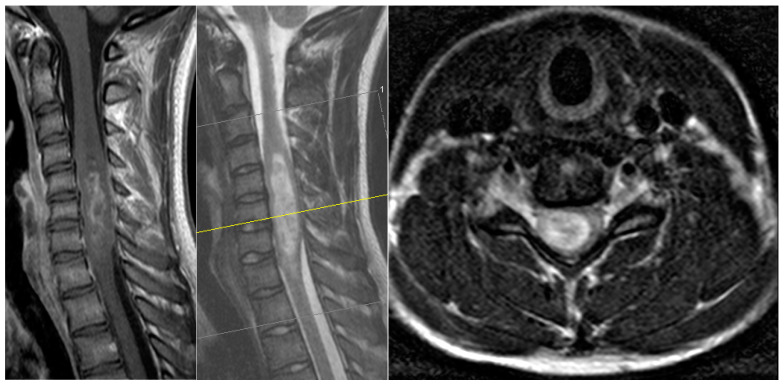
Magnetic resonance images showing the preoperative MRI scan of the patient with glioblastoma of the spinal cord at C4-Th1 (T2 and T1 with contrast enhancement, 1: sagittal view).

A 17-year-old male patient came to our emergency department with a subacute tetraparesis with accentuation on the right side. An MRI scan (Figure 2) revealed a large intramedullary lesion reaching to the upper cervical spinal cord and demonstrating irregular contrast enhancement, highly suspicious for a high-grade tumor. His neurological condition worsened overnight despite intravenous dexamethasone administration, and he developed a C5-level ASIA A quadriplegia by the next morning. We performed a microsurgical GTR of the lesion on the same day, initially hoping for a non-infiltrating tumor entity. The histopathology revealed a diagnosis of a glioblastoma. The patient did not recover from the quadriplegia. Due to the occurred respiratory insufficiency, early tracheotomy was performed 8 days later after the failed weaning. He was sent to neurological rehabilitation. Three months later, he was still quadriplegic, but no longer needed the tracheotomy. He passed away seven months after the surgery.

## 4. Discussion

### 4.1. Clinical Outcome

Surgery or at least a biopsy is usually inevitable during the treatment course of IMSCT. GTR is considered the best treatment strategy for the vast majority, as it benefits progression-free survival.

The risk for transient postoperative impairment is known to be significantly high. Previous research on patients with spinal ependymomas reported that more than 60% of patients experience neurological decline immediately after surgery [3,8,9]. In our case series, 77% of the patients developed new neurological deficits, respectively, resulting in a relevant functional decline in 40%, according to a deterioration in the McCormick scale. There is current evidence that the cervical location is an adverse predictive factor for functional outcomes after the surgery of IMSCT. In a large retrospective study reporting on ependymomas of the upper cervical spinal cord, Fei et al. reported on postoperative neurological impairment in 76% of patients and observed a subsequent slow recovery during the next 56 months, still rendering 21% of patients with permanent deterioration [10]. A similar trend of the preoperative, early, and late postoperative neurological status of their patients was reported by another series of intramedullary ependymomas, with a significant deterioration in McCormick scale in the early postoperative period and a recovery to the preoperative condition at the follow-up [11]. However, the difference in the patient’s neurological status was not accompanied by any relevant decline in functional disability, as reflected by the McCormick scale in our study.

### 4.2. Prognostic Factors for Neurologic Deterioration

We analyzed the predictive factors of postsurgical neurologic deterioration in the early and late follow-up periods. We found that the preoperative McCormick scale grade and the WHO grade were the strongest predictors of postoperative decline. This is consistent with several publications [12,13]. In accordance with our findings and previously conducted studies [10] observing a solid correlation of postoperative neurological decrease with the extent of preoperative disability, we recommend early surgery when patients’ neurological deficits are mild. As observed in our study, only a small proportion of patients improved their functional status compared with the status before surgery (5.7% in our series). However, 40% of the cases were clinically independent before surgery and therefore had no possibility of objective clinical improvement. According to this observation, the wait-and-see strategy, which would inevitably lead to a slow deterioration due to the tumor progression, appears unreasonable: first, retrieving the initial status quo is quite unlikely if the surgery is performed later on and second, the risk for further functional decline is rising during the waiting time.

The other significant criterion for poor neurologic outcomes at follow-up is the WHO grade. This could be explained by the higher neurologic deficits in patients with high-grade astrocytoma and metastases compared with those with ependymomas and hemangioblastomas at presentation. The infiltrating nature of these entities renders surgical treatment less safe. For this reason, the commonly accepted treatment strategy for these tumors is to refrain from GTR and to go for a sample collection via a biopsy to confirm the diagnosis and proceed with adjuvant treatment [5]. Furthermore, intramedullary higher-graded spinal cord gliomas have a poor prognosis despite surgery, radiation, and chemotherapy [5,14]. These treatment options can hardly prevent rapid neurological deterioration and poor overall survival of most patients. Future research on molecular genetics and targeted therapy for these tumors may bring new potential treatment options for these entities [15,16].

Intramedullary metastases occur mainly during the late stages of cancer and are frequently associated with leptomeningeal spread, reflecting an overall poor prognosis. However, surgical therapy may result in a temporary delay of further decline or even the improvement of neurological deficits in some cases. In contrast, the surgery’s only goal is spinal cord decompression by tumor mass debulking or reducing the associated syrinx [17,18].

Injury to the upper cervical spine carries the potential risk of respiratory failure, requiring mechanical ventilation, a prolonged ICU stay, prolonged weaning, an early tracheotomy, increased risk of pulmonary infections, and other associated complications [6]. The respiratory function cannot be monitored by conventional intraoperative monitoring. Nevertheless, though patients with IMSCT seem to be at risk for this adverse clinical course, the incidence appears relatively low. In our series, only three patients (8.6%) developed respiratory failure; in two cases (5.7%), this was clearly caused by upper spinal cord dysfunction. Although respiratory failure appears to be a relatively rare complication, we strongly recommend close monitoring for patients who underwent surgery of IMSCT of the upper spine during the first days after surgery and at least for 24 h due to possible life-threatening consequences.

### 4.3. Limitations

This study aimed to prove that respiratory issues caused by C4 paresis are not common after surgeries for intramedullary spinal cord tumors. While the results section did mention major complications, it is important to note that this retrospective study could not assess every complication, such as asymptomatic diaphragm (hemi-)paresis, due to a lack of standard postoperative assessments like chest X-rays or dynamic fluoroscopy. Furthermore, we did not assess the benefits of intraoperative ultrasounds (1).

Furthermore, the effects of cortisone during and after surgery may play an important role but were not routinely assessed in our retrospective study.

However, there were other limitations in the research, including a relatively small sample size since these types of tumors and those described are rare. Therefore, statistically significant conclusions about predictive factors for postoperative neurologic decline are difficult to be drawn with certainty from our cohort.

## 5. Conclusions

Patients with low-grade IMSCT of the upper cervical spine can maintain a good functional status after surgery during the long-term follow-up. However, they should be informed about the high likelihood of transient neurological deficits along with functional decline and temporary disability. Poor preoperative neurological condition and tumor malignancy may contribute to new neurologic deficits. The risk of respiratory deterioration is relatively low but should be considered for postoperative management and rehabilitation.

## Figures and Tables

**Table 1:** Clinical characteristics of 35 patients with intramedullary tumor of the upper cervical spine.

Characteristic	No. (%) of Mean ± SD
Male	20 (57%)
Mean age overall in yrs	45 ± 16
Histopathology	
Ependymoma (WHO°1–2)	22 (62.8%)
Astrocytoma (WHO°2–4)	4 (11.4%)
Hemangioblastoma WHO 1	6 (17.1%)
Metastases	2 (5.7%)
Other	1 (2.86%)
Patients with neurofibromatosis type 2	4 (11.4%)
Median duration of symptoms in month	3 ± 48
Mean tumor vol in cm^3^	1.86 ± 2.58
Presence of syringomyelia	8 (23%)
Extent of tumor resection	
Gross total resection	25 (71.5%)
Subtotal resection	7 (20.0%)
Biopsy	3 (8.6%)

**Table 2:** Preoperative symptoms in patients with intramedullary tumor of the upper cervical spine.

Symptom	No. (%)
Sensoric deficit	41.7
Motoric deficit	38.9
Pain	25.0
Gait ataxia	19.4

**Table 3:** Results of the neurological outcome in all patients. = means no worsening.

Preoperative Deficit	Postoperative Deficit	New Motor Deficit	New Sensory Deficit	Motor Deficit at FU	Sensory Deficit at FU
gait ataxia, hypesthesia sub C5	accentuation of hypesthesia	no	yes	No	Yes
ASIA D	hemiplegia	yes	no	Yes	no
intermittent urinary incontinence	arm paresis MMT 3/5	yes	yes	Yes	no
hypesthesia	gait ataxia	no	yes	lost to FU	lost to FU
mild tetraparesis MMT 4/5	aggraviation of distal arm paresis	yes	yes	No	no
no deficit (neck pain)	hypesthesia	no	yes	No	yes
gait ataxia	mild arm paresis	yes	no	Yes	no
tingling sensation	aggraviation of tingling sensation/hypesthesia	no	yes	no	yes
no deficit (shoulder pain)	mild arm paresis, hypesthesia	yes	yes	no	no
hemiparesis	aggraviation of hemihypesthesia	no	yes	no	yes
hemihypesthesia	=	no	no	no	no
no deficit (vertigo)	hemihypesthesia	no	yes	lost to FU	lost to FU
mild arm paresis	new hemihypesthesia	no	yes	no	no
ASIA C	aggraviation of motor deficit	yes	no	lost to FU	lost to FU
gait ataxia	aggraviation of gait ataxia	no	yes	no	no
no deficit (neck pain)	hemiparesis	yes	yes	yes	yes
no deficit (neck pain)	hemiparesis	yes	yes	yes	yes
gait ataxia, hypesthesia sub C6	ASIA C	yes	yes	lost to FU	lost to FU
no deficit (neck and arm pain)	diffuse hypesthesia	no	yes	no	no
no deficit (neck and arm pain)	diffuse hypesthesia	no	yes	no	yes
gait ataxia	mild arm paresis	yes	yes	no	yes
fine motor disability	=	no	no	yes	yes
allodynia	=	no	no	no	no
mild arm paresis	accentuation of arm paresis	yes	no	yes	no
mild arm paresis	ASIA D	yes	yes	no	no
ASIA B	ASIA B	no	no	no	no
no deficit (neck and arm pain)	hemihypesthesia	no	yes	no	yes
tingling sensation	hemiparesis	yes	yes	yes	yes
no deficit (neck and arm pain)	=	no	no	no	no
hypesthesia	=	no	no	no	no
gait ataxia, arm paresis	accentuation of arm paresis	yes	yes	yes	yes
hemihypesthesia	accentuation of hemihypesthesia	no	yes	no	no
severe hemiparsis	=	no	no	lost to FU	lost to FU
ASIA A	ASIA A	no	no	no	no
severe hemiparsis	accentuation of severe hemiparesis	yes	no	lost to FU	lost to FU
		15	22	9	12
		43%	63%	31%	41%

**Table 4:** Results of the multivariate regression analysis predicting early neurological impairment. * *p* < 0.05; *** *p* < 0.001.

Variable	*p* Value
Age	0.518
Sex	0.257
Tumor vol	0.070
Duration of symptoms	0.064
Preop McCormick score	<0.001 ***
WHO grade	0.045 *
Surgical approach	0.483
Extension of resection	0.415
Syrinx	0.335
Loss of potentials in IONM	0.016 *

**Table 5:** Results of the multivariate regression analysis predicting late neurological impairment. * *p* < 0.05; *** *p* < 0.001.

Variable	*p* Value
Age	0.497
Sex	0.433
Tumor vol	0.151
Duration of symptoms	0.373
Preop McCormick score	<0.001 ***
WHO grade	0.009 *
Surgical approach	0.212
Extension of resection	0.796
Syrinx	0.493
Loss of potentials in IONM	0.202

## Data Availability

The datasets used and/or analyzed during the current study are available from the corresponding author on reasonable request.

## References

[1] Samartzis D., Gillis C.C., Shih P., O’Toole J.E., Fessler R.G. (2015). Intramedullary Spinal Cord Tumors: Part I-Epidemiology, Pathophysiology, and Diagnosis. Glob. Spine J..

[2] Butenschoen V.M., Schwendner M., Hubertus V., Onken J., Koegl N., Mohme T., Maurer S., Boeckh-Behrens T., Eicker S.O., Thomé C. (2023). Preoperative angiographic considerations and neurological outcome after surgical treatment of intradural spinal hemangioblastoma: A multicenter retrospective case series. J. Neurooncol..

[3] Wostrack M., Ringel F., Eicker S.O., Jagersberg M., Schaller K., Kerschbaumer J., Thomé C., Shiban E., Stoffel M., Friedrich B. (2018). Spinal ependymoma in adults: A multicenter investigation of surgical outcome and progression-free survival. J. Neurosurg. Spine.

[4] Hersh A.M., Antar A., Pennington Z., Aygun N., Patel J., Goldsborough E., Porras J.L., Elsamadicy A.A., Lubelski D., Wolinsky J.P. (2022). Predictors of survival and time to progression following operative management of intramedullary spinal cord astrocytomas. J. Neurooncol..

[5] Butenschoen V.M., Hubertus V., Janssen I.K., Onken J., Wipplinger C., Mende K.C., Eicker S.O., Kehl V., Thomé C., Vajkoczy P. (2021). Surgical treatment and neurological outcome of infiltrating intramedullary astrocytoma WHO II-IV: A multicenter retrospective case series. J. Neurooncol..

[6] Galeiras Vazquez R., Rascado Sedes P., Mourelo Farina M., Montoto Marques A., Ferreiro Velasco M.E. (2013). Respiratory management in the patient with spinal cord injury. Biomed. Res. Int..

[7] McCormick P.C., Torres R., Post K.D., Stein B.M. (1990). Intramedullary ependymoma of the spinal cord. J. Neurosurg..

[8] Gembruch O., Chihi M., Haarmann M., Parlak A., Oppong M.D., Rauschenbach L., Michel A., Jabbarli R., Ahmadipour Y., Sure U. (2021). Surgical outcome and prognostic factors in spinal cord ependymoma: A single-center, long-term follow-up study. Ther. Adv. Neurol. Disord..

[9] Hussain I., Parker W.E., Barzilai O., Bilsky M.H. (2020). Surgical Management of Intramedullary Spinal Cord Tumors. Neurosurg. Clin. N. Am..

[10] Fei X., Jia W., Gao H., Yang C., Li D., Qian Z., Han B., Wang D., Xu Y. (2021). Clinical characteristics and surgical outcomes of ependymomas in the upper cervical spinal cord: A single-center experience of 155 consecutive patients. Neurosurg. Rev..

[11] Takami T., Naito K., Yamagata T., Ohata K. (2015). Surgical management of spinal intramedullary tumors: Radical and safe strategy for benign tumors. Neurol. Med. Chir..

[12] Bostrom A., Kanther N.C., Grote A., Bostrom J. (2014). Management and outcome in adult intramedullary spinal cord tumours: A 20-year single institution experience. BMC Res. Notes.

[13] Lee S.M., Cho Y.E., Kwon Y.M. (2014). Neurological outcome after surgical treatment of intramedullary spinal cord tumors. Korean J. Spine.

[14] Houten J.K., Cooper P.R. (2000). Spinal cord astrocytomas: Presentation, management and outcome. J. Neurooncol..

[15] Azad T.D., Jiang B., Bettegowda C. (2019). Molecular foundations of primary spinal tumors-implications for surgical management. Ann. Transl. Med..

[16] Grady C., Melnick K., Porche K., Dastmalchi F., Hoh D.J., Rahman M., Ghiaseddin A. (2022). Glioma Immunotherapy: Advances and Challenges for Spinal Cord Gliomas. Neurospine.

[17] Payer S., Mende K.C., Westphal M., Eicker S.O. (2015). Intramedullary spinal cord metastases: An increasingly common diagnosis. Neurosurg. Focus..

[18] Wostrack M., Pape H., Kreutzer J., Ringel F., Meyer B., Stoffel M. (2012). Surgical treatment of spinal intradural carcinoma metastases. Acta Neurochir..

